# Mobile survey engagement by older adults is high during multiple phases of the COVID-19 pandemic and is predicted by baseline and structural factors

**DOI:** 10.3389/fdgth.2022.920706

**Published:** 2022-08-23

**Authors:** Federica Klaus, Elizabeth Peek, Avery Quynh, Ashley N. Sutherland, Divya Selvam, Raeanne C. Moore, Colin A. Depp, Lisa T. Eyler

**Affiliations:** ^1^ Department of Psychiatry, UC San Diego, La Jolla, CA, United States; ^2^ VA San Diego Healthcare System, Mental Illness Research, Education, and Clinical Center (MIRECC), La Jolla, CA, United States

**Keywords:** ecological momentary assessment, survey, adherence, withdrawal, stay-at-home, mobile phone, older adults, pandemic

## Abstract

Digital surveys, such as mobile phone ecological momentary assessment (EMA), bear the potential to assess and target individual wellbeing in a personalized, real-time approach and allow for interaction in situations when in-person contact is not possible, such as during the coronavirus pandemic. While the use of digital technology might especially benefit research in older adults who find themselves in circumstances of reduced mobility, little is known about their barriers to adherence. We investigated baseline and structural factors that predict study withdrawal and adherence from daily smartphone EMA self-report surveys in the StayWELL Study. The StayWELL study is a longitudinal, observational study on the relationship between social restrictions during the coronavirus pandemic and mental well-being in 95 community-dwelling older aged adults (67–87 years) who were participants in a randomized clinical trial using EMA. Withdrawal was associated with less research staff changes and less likely in participants that reached the study mid-point. No baseline characteristics predicted withdrawal. Main reasons for withdrawal were communication issues, i.e. staff not being able to contact participants. We found an adherence rate of 82% and no fatigue effects. Adherence was predicted by education status, study participation duration, reaching the study midpoint and time between study start and enrollment. COVID infections or supporting people in the household was not related to adherence. To conclude, it is feasible to conduct an EMA study in older people without impacting engagement during a pandemic. Furthermore, personal characteristics and smartphone operating system (Android vs. iOS) used did not relate to engagement, allowing for a broad distribution of digital health technologies. Our study adds information on single predictive variables relevant for adherence and withdrawal from EMA smartphone surveys in older people that can inform the design of future digital EMA research to maximize engagement and reliability of study results.

## Introduction

Ecological Momentary Assessment (EMA) repeatedly samples participants' current mood, behaviors, and sense of wellbeing in real time in their natural environments (1, 2). Surveys delivered multiple times daily *via* mobile phone apps with text messaging reminders can capture self-reports in real-time and minimize recall bias while preserving ecological validity (1, 2). However, to best capture momentary feelings and behaviors, it is necessary for participants to answer as many surveys as possible and to stay in the study throughout its duration. Missing data due to low EMA adherence rate and, as an extreme form, study withdrawal, are important since they lead to low statistical power, response bias, and increased cost to the researchers (3). It is important to understand typical rates of withdrawal and adherence in order to power future studies to discover factors that predict adherence and withdrawal in order to devise protocols that maximize participation.

In the general population, one predictor of high overall adherence rates is high adherence in the early phase of the protocol (3). Contextual factors, such as time of day, may play a role in missing survey reminders whereas increased training on how to use EMA is correlated with higher adherence levels (4). Some research suggests that older age, healthy mood and affect were correlated with higher levels of adherence (4–9).

Some early research has been done using EMA with older adults; but the scope of the literature is narrow. This can partially be attributed to the fact that in 2015, only 27% of American older adults owned a smartphone; however smartphone ownership among older adults is growing rapidly with 83% of those 50–64 years old and 61% of those 65 years and older owning a smartphone in the US in 2021 (10–13). While older adults are more reluctant to use new technology, they are more likely to utilize new technologies when they understand the benefits (14, 15). A review on EMA in aging research reported a general adherence rate of over 80% in most studies assessed (16), which is higher compared to younger adults, where a survey adherence rate of 75% with a withdrawal rate of 15% was reported (5, 17). One study used EMA over a 14-day burst to look at daily activities and neurocognitive health in 103 older American adults and observed an adherence rate of 91% (18). Another study looked at EMA engagement for African American older adults, a group that was expected to be more wary of EMA and see it as surveillance; however they also had high adherence rates of on average 92%–98% and a withdrawal rate of 9% over the whole study (19). Graham et al. examined American users of a digital healthcare platform with multiple interfaces, and found that on an aggregate level, older users (65 years and older) utilized the app more than younger users (35–64 years) (20). In a sample of older participants (aged 50–70 years), 95 older adults were sent six surveys per day for a week and had a 91.5% response rate (21). In a study measuring older adults' (60–98 years) physical activity using EMA, an adherence of 92% and withdrawal rate of 2% was observed (8).

To understand the reasons for withdrawal from an EMA study and what influences withdrawal from and adherence to EMA surveys, more detailed information on personal baseline factors and study structural factors are needed. In the previously cited studies, systematic information on reasons for study withdrawal in older adults are rarely reported, with one study mentioning withdrawal due to medical emergency or participant burden in older adults (8) and another describing as a primary withdrawal reason that participants did not fully understand what they were supposed to do (22). Moderators of adherence are reported for some, but not all studies and focus mainly on personal factors, such as age, sex, relationship status, residence, number of people in residence, education, employment status, income, and location. Most studies do not find an association between personal baseline characteristics and study adherence (8, 19). One study in older adults (50–70 years) observed higher adherence in female participants aged 50–59 years (93.3%) vs. male participants of the same age (84.5%), but overall adherence levels were above the recommended 80% (21). Another study in older people reported no relation of age to completion or response rate, but found that older participants were more likely to report not being alerted to surveys and that issues with survey alerts were independently related to Android operating system (23). The same study also observed higher response rates among iOS vs. Android users (23). However, information on other structural factors specific to older adults' adherence to EMA studies is sparse. One study reported a higher likelihood of missing an EMA survey in the afternoon compared to the morning in healthy older adults, providing information on time-varying factors (8).

The above-described studies provide evidence that withdrawal from EMA studies among older adults is generally low and EMA adherence is generally high in typical settings. However, it is unclear whether the same would be seen during a global pandemic such as that caused by COVID-19. Due to social distancing measures during the pandemic, studies suggest that older adults increased their technology consumption. One study showed that older adults used technology to connect socially and two-thirds of participants learned a new communication technology (24). Another study showed that 73% of German nursing homes self-reported increased opportunities for residents to connect virtually (25). Another study demonstrated that there was an increase in older adults who ordered groceries using mobile delivery (26). This shows that there was increased use of digital technology during the pandemic among older people. Additionally, there was an increase in using EMA methods during the pandemic with researchers being unable to conduct in-person visits due to social distancing; however, there is limited literature about adherence levels for older adults in this context. Most recently, a study in 47 older adults (age 45–78 years) during the pandemic sent six surveys per day for a week and had a 84% vs. 54% completion and a 64% vs. 54% response rate among experienced vs. inexperienced EMA users (23).

We created the Stay-at-home Wellness EMA in Late Life (StayWELL) Study to understand the wellbeing of older adults during the pandemic. StayWELL enrolled a well-characterized sample of older adults (>65 years) who had previously completed a randomized controlled trial which included EMA sampling and collection of detailed demographic and psychological assessments. As a completely virtual, longitudinal study, StayWELL collected self-report data on mental wellbeing and daily activities, using online questionnaires and EMA *via* mobile surveys, throughout the pandemic. EMA data was collected during two 2-week bursts of assessments in Summer/Autumn 2020 and in Summer of 2021. The data from this study therefore provides a unique opportunity to assess factors that predict withdrawal and adherence to EMA in older adults over the course of a seventeen-month period, beginning during the sudden and long-term shutdown of normal behavior and routines and a forced shift to digital technology. Given the lack of longitudinal studies on older adults during the coronavirus pandemic, our study provides the opportunity to capture a wide breadth and depth of data on potential predictors of EMA study adherence and study withdrawal factors of older adults in situations of reduced mobility due to pandemic-related social restrictions.

We investigated study withdrawal and adherence to daily EMA surveys in the StayWELL study. The first aim was to explore reasons for withdrawal and if any personal baseline factors predicted withdrawal in older adults, including age, gender, race/ethnicity, employment, residence, number of people in residence, education, location, generations in the household, how often the house was left pre-pandemic and previous participation in an active treatment arm of the prior study randomized controlled trial (vs. a control condition). Further, we examined if study structural factors, including study research staff turnover, smartphone operating system, time elapsed between study start and enrollment and reaching the study mid-point predicted withdrawal.

The second aim was to examine if any personal baseline or study structural factors predicted adherence—per burst—in older adults, based on the same personal and structural factors explored in relation to withdrawal. We hypothesized that adherence will be predicted by age and gender with highest adherence in younger women based on specific findings on older adults (21).

The final aim was to understand how the rate of completed EMA surveys from the first two bursts (burst 1 and 2; administered before significant lifting of restrictions, i.e. stay-at-home and social distancing order, in Summer/Fall 2020) compared to the last two bursts (burst 3 and 4; after lifting of restrictions in Summer of 2021). We hypothesized that adherence would be significantly less during burst 3 and 4 due to participant's reengaging in activities outside the home.

## Materials and methods

### Participants and recruitment

Ninety-five community-dwelling older aged adults (67–87 years) were included in the StayWELL study. All had previously participated in the Mindfulness, Education, and Exercise (MEDEX) study (27) and were concurrently enrolled in an extension trial. The MEDEX study was a now-concluded 18-month randomized controlled trial to assess the effects of in-person interventions, which included three active treatments, i.e. mindfulness-based meditation, exercise and their combination, and one active control group, i.e. health education, on cognition in older adults. MEDEX also included daily EMA assessments on study-provided tablets of self-reports (such as positive and negative affect) during four 10-day periods for all groups. Most participants who completed the in-person study then continued to take part in the extension of the randomized trial, where participants received once per month a virtual booster session of the same intervention that they had received in the previous trial. The extension of MEDEX started in October 2018 and is ongoing planned until September 2023. Participants were recruited by MEDEX staff during the monthly virtual booster sessions and when they received MEDEX study related information *via* mail on a rolling basis. After receiving advertisement about the StayWELL Study, 124 MEDEX study participants contacted the StayWELL study team and 95 of these participants decided to participate after hearing what study participation entailed. Couples were allowed to participate in the study and enrolled occasionally (approximately 2 couples).

Inclusion criteria were previous participation in the MEDEX study and current enrollment in the MEDEX extension study (St. Louis or San Diego), and the possession of a mobile device (Android or iOS operating system) with touch screen and internet access. All procedures were approved by UCSD's Institutional Review Board before protocol implementation, and all participants provided oral informed consent.

### Measures and procedures

#### Study design

The fully virtual StayWELL study began in June 2020 and was completed in October 2021.

**Figure 1:**
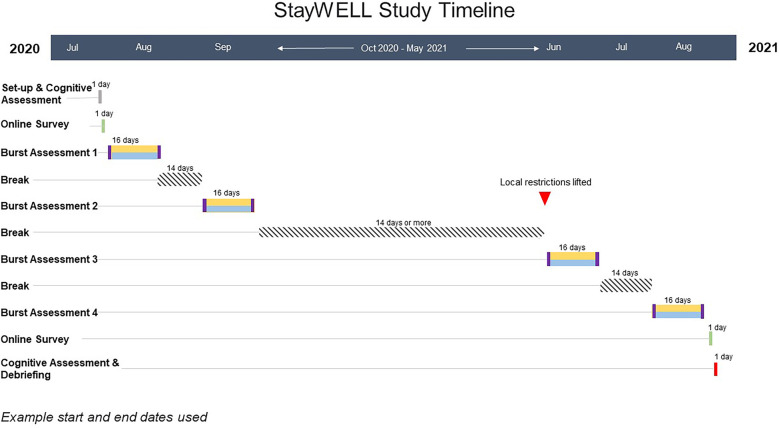
Exemplary timeline of StayWELL study design.

One study visit at the beginning and end of the study included a Set-up/Final Call and cognitive assessment, of which the data is not analyzed at this point, and direction to an online questionnaire. In-between, four EMA bursts, which lasted each 16 (14 + 2)-days, with a long break in-between burst 2 and 3 (mid-study) took place (See Figure 1). Due to the rolling advertisement with participants contacting the study staff at their will, time between study start and actual study enrollment varied between participants.

Self-report data, including mental wellbeing and daily activities, was collected using online questionnaires and EMA *via* mobile surveys throughout the pandemic. All study visits were conducted *via* videoconferencing using the software Zoom or *via* phone at the beginning and end of the study and baseline information (demographics etc.) was assessed using the online questionnaire at the beginning and end of the study. The number of available staff or volunteer research associates (research assistants (RA's)) did oscillate due to the unexpected long duration of the study with the restrictions during the pandemic remaining in place longer than anticipated. When this longitudinal study was conceived, a duration of the pandemic of a few weeks and therefore a study duration of maximum half a year was generally expected, which was proven wrong by the prolonged nature of the pandemic. These events led to unexpected personal circumstances that were reflected in the availability of RA's. At the beginning of the study, each participant was assigned to one of the available ten RAs. During the study, some participants were re-assigned to other RA's due to the reduced availability or change of position of some (due to personal and educational duties). Short communication by RA's (e.g. for confirmation of appointments or check-in visits) was mostly conducted using Skype phone calling, Google voice calling or Google voice texting or email. Longer visits/trouble-shooting visits were conducted by sending a Zoom link to the participants *via* email and then conducting a video call using Zoom. Participants received a $30 Amazon gift card at the conclusion of the study. If they complete greater than 85% of the daily EMA surveys, they received a bonus of $20 in the form of an additional gift card.

#### Smartphone set-up visit

After giving initial information to the potential study participants and obtaining consent *via* a phone call, an RA was assigned to each participant. The RA contacted the participant *via* Zoom (video call) and conducted a 1-hour long set-up visit, during which the participants were guided to install all necessary applications on their own mobile devices and participants were provided an individualized live tutorial delivered by an RA that also used recorded video sequences of how to install the apps on their mobile phone (iOS or Android specific) and how to complete EMA surveys. Further, they were given, *via* email, a written and illustrated document explaining how to use the apps on their smartphone. At the end of the setup call, the participants practiced an example EMA survey that came through to their mobile device. Participants were provided contact information in the event they experienced technological difficulties and were contacted if a drop in completed surveys suggested difficulties. The platform and application mEMA (Illumivu) was used for the smartphone-based ecological momentary assessment and could be downloaded from the respective AppStores by the participants on their mobile devices.

#### EMA bursts

Four 16-day total mobile burst assessments took place (bursts 1–4). The first two bursts started after enrollment with an approximate gap of 2 weeks in-between (depending on participant's availability) and were completed in Summer/Fall 2020. The third burst was planned to occur upon significant lifting or pandemic-related social restrictions and started in Summer/Fall 2021 upon lifting of restrictions and the fourth burst followed after an approximate 2-week gap.

Each burst started and ended with a longer survey (20 min, scheduled to arrive in the morning) asking about thoughts, behaviors and events related to well-being and the coronavirus pandemic over the past 2 weeks on the first and last day. Each burst then included 14 days of twice-daily brief momentary surveys (5–10 min long, scheduled in morning and evening randomly within a 1-hour time window according to participant's preference). The brief daily surveys asked about wellbeing, mood, compassion, empathy, social isolation, mindfulness, resilience, and loneliness and behavior in the moment. A pop-up notification and a sound alert (if not set to mute) was sent that reminded the participant to take the surveys. The surveys started at random times (15 min, 30 min, 45 min or 60 min apart from the last survey) within the specified 1-hour window and were available to answer until 1 h after the notification first appeared (see Figure 1).

All participants were contacted mid-study to enhance retention and explain the protocol for the remaining bursts. The final timing of assessments differed from what was anticipated at study start due to the protracted course of the pandemic. Thus, although four bursts had been planned all along, the total time in the study was much longer than expected by participants when they enrolled. In addition to beginning and end-of study contacts, RA's contacted participants a week in advance before each burst started, at the start date of each burst, during the burst if needed, at the end of each burst and during the longer mid-study break to remind participants that the study was still ongoing.

### Data analysis

#### Time frame and variables used

Number of surveys completed out of the 4 × 14-days short daily surveys in the morning and evening were analyzed in this study and morning and evening surveys were collapsed into a daily average.

Adherence was calculated as percentage of surveys validly answered per burst and per burst category (burst 1/2 and burst 3/4 were collapsed into burst categories before and after lifting of stay-at-home restrictions).

Analysis of adherence was conducted in all participants that contributed EMA data. Analysis of withdrawal included all participants that initially consented to the study. Only data from participants who completed burst 1 and/or 2 and burst 3 and/or 4 were used for the analysis of adherence differences between burst.

Demographic variables were collected at baseline. The original choices for people in the same household (number of people) were collapsed into categories: (I live alone, 1 person, 2 or more people). Information about COVID-19 diagnosis and support given to family members was derived from the longer survey at the end of each burst covering the past 2 weeks.

Quantitative assessment of withdrawal reasons was performed by classifying the reasons given for withdrawal by the participants into the following categories: participant was hard to reach/communication issues, unknown/reason not given, no longer interested/too busy, technical difficulties, personal/unspecified and health reasons. Qualitative assessment of feedback given by participants is based on the notes taken of the conversations by the RAs who received the note of withdrawal by the participant.

#### Statistical analysis

Statistical analyses were computed with SPSS version 25 (IBM Corp., SPSS Inc., Chicago IL, USA). Demographic variables were compared using chi-square, Mann-Whitney U or two-tailed t-tests, as appropriate. Generalized linear mixed model analyses were conducted with subject ID as random effects in all analyses. Level of significance was set at *p *< 0.05. Multiple testing was accounted for using the false discovery rate (FDR) (28). Data are presented as mean and standard deviation (SD) if not noted otherwise. Data of all participants who consented to the study were analyzed for withdrawal analyses. Only data from participants where EMA survey were set up were analyzed in analyses investigating adherence.

Specifically, the following methods were applied for each study aim:

1) To assess predictors of and reasons for withdrawal, data of all participants that consented to study participation were entered in the analysis. Separate models with baseline predictors as fixed effects were ran with age, gender, race/ethnicity, employment status, type of residence, education, location (San Diego vs. St. Louis), number of people living in the same residence and assignment to the MEDEX treatment group as well as generations in the household and how often the house was left (before the pandemic).
  To assess the structural variables, number of changes of RA, operating system, time elapsed between study start to enrollment and reaching burst 3 (i.e. the second half of the study/study mid-point) were used as fixed effects. All significant predictors were then combined into one model to investigate potential dependencies. Spearman correlations due to non-normal distribution of date variables were used to assess relationships of number of RA changes with study participation duration, since participants that were longer in the study might have experienced more RA changes. A multivariate model with significant predictors and study participation duration was used to account for dependency of RA change on study participation duration length.

2) To assess predictors of adherence, data of all participants that had EMA surveys set up were entered in the analysis. Separate models with burst and each baseline predictor as fixed effects were used with age, gender, race/ethnicity, employment status, type of residence, education, location (San Diego vs. St. Louis), number of people living in the same residence and assignment to the MEDEX treatment group as well as generations in the household and how often the house was left (before the pandemic). To assess the structural variables, number of changes of RA, operating system, time elapsed between study start to enrollment and reaching burst 3 (i.e. the second half of the study) were used in separate models as fixed effects in addition to burst. Correction for multiple testing using FDR was applied (denoted as adjusted p (adj.p)). Finally, all individual variables that remained significant after FDR correction were entered into one model to investigate potential dependencies.
  To follow up on a hypothetical moderation of the relationship between people living in residence and adherence by COVID-19 infections in the family and giving support to family members, two separate models were calculated. Fixed effects were people in the residence and support given to family members, or people in the residence and COVID-19 infections of family members and their respective interaction terms.
  To predict adherence per burst based on structural factors, separate mixed model analyses were used with burst, number of changes of RA, operating system, time elapsed between study start to date of individual study enrollment, and study participation duration as fixed effects. Spearman correlations due to non-normal distribution of date variables were used to assess relationships of number of RA changes with time elapsed between study start and enrollment and with study participation duration, since participants that were longer in the study might have experienced more RA changes.

3) To compare the rate of completed EMA surveys from the first two to the last two bursts within those participants that contributed data to burst 1 and/or 2 and to burst 3 and/or 4, mixed model analyses were used with burst and burst category as fixed effects respectively.

## Results

### Sample characteristics

Ninety-five participants enrolled in the study and 47 participants completed study procedures ((49.5%, 67–87 years) whereas the other 48 participants (50.5%, 67–83 years) withdrew.

The mean (SD) age of all enrolled participants at baseline was 74 (4.3) years, 78% were women, and the mean (SD) education was 16.6 (2.0) years. The racial distribution was 84% White, 8% Black/African American, 4% Asian, and 3% More than One Race; Hispanic participants were 5% of the sample (See Table 1 for details.)

**Table 1:** Demographics of study participants who withdrew and who completed the study.

	Participants who withdrew (*n* = 48)		Participants who completed the study (*n* = 47)		Test statistics	
	Mean (range) or n (%)	SD	Mean (range) or n (%)	SD	*t/χ* ^2^ */U*	*p*
Age (years)	73.8 (67-83)	3.9	73.7 (67-87)	72.3	*t = *−*1.5*	0.9
Gender (F (%))	40 (83%)		34 (72%)		*χ*^2^* = 1*.*7*	0.2
Education (years)	16.6	2.4	16.7	1.6	*U = 1075*	0.9
Race (*n* (%))			* *		*χ*^2^* = 3*.*4*	0.4
White	39 (81%)		41 (87%)			
Black/African American	6 (13%)		2 (4%)			
Asian	1 (2%)		3 (6%)			
More than one Race	2 (4%)		1 (2%)			
Ethnicity (Non-Latino-Hispanic (%))	44 (91%)		46 (97%)		*χ*^2^* = 1*.*8*	0.18
Employment status (Retired (%))	27 (56%)		42 (89%)		*χ*^2^* = 3*.*1*	0.4
Number of people living in same household (0/1/2 or more)	21%/40%/6%		26%/66%/8%		*χ*^2^* = 2*.*6*	0.6
Number of generations living in same household	0.2	0.4	0.4	0.0	*U = 1210*	0.4
How often house was left per day before pandemic (days per week)	4.3	0.9	4.0	0.9	*U = 1733*	0.1
COVID19 Diagnosis of family member (burst 1/2/3/4)	31%/9%/0%/0%		9%/20%/16%/18%			
Support to family members given (burst 1/2/3/4)	23%/27%/0%/0%		30%/29%/35%/23%			
Location (San Diego (%))	22 (45%)		27 (57%)		*χ*^2^* = 1*.*3*	0.3
Medex intervention group			* *		*χ*^2^* = 3*.*4*	0.3
MBSR (mindfulness-based meditation)	11 (23%)		16 (34%)			
Exercise	12 (25%)		14 (30%)			
MBSR + Exercise	18 (38%)		10 (21%)			
Health Education (comparison group)	7 (15%)		7 (15%)			
Number of research assistant (RA) changes	0.3	0.6	0.7	0.7	*U = 1885*	***p* < 0.001**
Operating System (Android/iOS (%))	18 (38%)/30 (62%)		16 (34%)/31 (66%)		*χ*^2^* = 1*.*2*	0.7
Bursts completed (0/1/2/3/4)	11/12/16/0/0		1/2/3/10/31			
Last completed burst (0/1/2/3/4)	20/12/15/1/0		1/2/3/3/38			
Withdrawal timing (until during burst 2/between burst 2 and 3/during or after burst 3)	27/19/2		–		*–*	–
Study participation duration (years)	0.5	0.4	1.1	0.2	*U = 1336*	***p* < 0.001**
Time elapsed between study start and date of enrollment (months)	1.5	0.9	1.5	0.8	*U = 2226*	0.8

Significant results with *p*-values < 0.05 are bolded.

*χ*^2^, Chi-square; U, Mann-Whitney U; t, two-tailed t-test.

Among the participants that completed the study, 42 participants contributed data to burst 1 and/or 2 and to burst 3 and/or burst 4 (See **Supplementary Table S1** for details.).

### Participants who reached the study mid-point were less likely to withdraw after that time-point

Analysis of withdrawal were conducted in the whole dataset containing 95 participants. Statistical details on estimates for each level of predictor can be found in the **Supplementary information**.

Of all participants that withdrew, 46 participants withdrew before burst 3 and 2 participants withdrew after burst 3. See Table 2 for exact timing of withdrawal. Main reasons for withdrawal were staff- participant communication issues (31%), not enough time for the study (21%) and no reasons reported (21%). Technical difficulties accounted for 15% of withdrawals, partially contrary to our initial hypothesis (see Table 3).

**Table 2:** Timing and reason for withdrawal.

Timing of withdrawal	Reason for withdrawal						
	no longer interested/too busy	technical difficulties	personal/unspecified	health reasons	hard to reach/communication issues	unknown/reason not given	Total
before burst 1	0	2	1	0	5	4	**12**
during burst 1	1	1	0	0	1	0	**3**
after burst 1 before burst 2	2	2	2	0	3	1	**10**
during burst 2	1	1	0	0	0	0	**2**
after burst 2 before burst 3	5	1	2	1	5	5	**19**
during burst 3	0	0	0	0	0	0	**0**
after burst 3 before burst 4	1	0	0	0	1	0	**2**
during burst 4	0	0	0	0	0	0	**0**
Total	**10**	**7**	**5**	**1**	**15**	**10**	

Significant results with *p*-values < 0.05 are bolded.

**Table 3:** Reasons for study withdrawal.

Reason for withdrawal	Count	Percent per total withdrawals/ total participants
hard to reach/communication issues (participant could not be contacted)	15	31% / 15.8%
unknown/reason not given	10	21% / 10.5%
no longer interested/too busy	10	21% / 10.5%
technical difficulties	7	15% / 7.4%
personal/unspecified	5	10% / 5.3%
health reasons	1	2% / 1.1%

*Baseline structural characteristics* did not predict withdrawal, specifically withdrawal was not predicted by gender (*F(*1,93) = 1.6, *p *= 0.21), age (*F*(1,93) = 0.022, *p *= 0.88), race (*F*(3,91) = 0.99, *p *= 0.40), ethnicity (*F*(1,93) = 1.56, *p *= 0.21), employment status (*F*(3,75) = 0.01, *p *= 0.99), residence type (*F*(2,76) = 0.23, *p *= 0.79), education status (*F*(1,91) = 0.06, *p *= 0.81), location (*F*(1,93) = 1.25, *p *= 0.27), generations in household (*F*(2,76) = 0.05, *p *= 0.95), how often the house was left before the pandemic (*F*(3,75) = 1.18, *p *= 0.32), number of people living in residence (*F*(2,76) = 0.18, *p *= 0.84) or MEDEX intervention group (*F*(3,91) = 1.05, *p *= 0.37).

*Structural factors* significantly related to withdrawal were number of changes of RA's (*F*(2,92) = 6.9, *p *= 0.002) and making it beyond the mid-study point, i.e. reaching burst 3 (*F*(1,93) = 34.5, *p *< 0.001), which remained significant after adjusting for multiple testing (*adj.p *= 0.003 and *adj.p *< 0.001, respectively). Completing the study was associated with having 1 RA change vs. 0 (*p *< 0.001, odds ratio = 0.14) and with reaching burst 3 (*p *< 0.001, odds ratio = 0.006). There was no relation of withdrawal with operating system (*F*(1,93) = 0.12, *p *= 0.73) and time elapsed between study start and enrollment (*F*(1,93) = 0.07, *p *= 0.79). When combining the two significant predictors into one model, the relationship of both RA changes (*F*(2,91) = 3.23, *p *= 0.04) and reaching burst 3 (*F*(1,91) = 31.1, *p *< 0.001) to withdrawal remained significant, with having no vs. 1 or 2 RA changes predicting withdrawal (*p *= 0.04, odds ratio = 0.15 and *p *= 0.03, odds ratio = 0.09 respectively). Because there were significant positive correlations of number of RA changes with study participation duration (*r_s_(*95) = 0.31, *p* = 0.003), we also examined a model with additionally study participation duration included. In that model, number of RA changes was no longer significant (*F*(2,90) = 1.53, *p *= 0.22), while reaching burst 3 remained a significant predictor of withdrawal (*F*(1,90) = 22.7, *p *= 0.04), indicating that RA changes are dependent on the other two predictors.

*Adherence* to EMA surveys was not significantly associated with study withdrawal (*F*(1,72) = 3.59, *p* = 0.06), the direction of the trend-level relationship was such that poor adherence was associated with greater likelihood of withdrawal (odds ratio = 0.98, 95% confidence interval [0.96, 1.00]).

### Adherence was predicted by education status, study participation duration, time between study start and enrollment and reaching the second half of the study

Analysis of adherence was conducted in all participants that contributed EMA data (*n* = 74). Statistical details on estimates for each level of predictor can be found in the **Supplementary information**.

Among *baseline characteristics*, education status significantly predicted adherence after correction for multiple testing (*F*(1,206) = 6.27, *p *= 0.013, adj.*p *= 0.04)), with more years of education being associated with higher adherence. The significant relation of adherence to number of people living in residence (*F*(2,192) = 4.48, *p *= 0.01 (adj.*p *= 0.05), living with 2 people or more vs. living alone was associated with poorer adherence), participation in the MEDEX active treatment groups (*F*(3, 210) = 3.96, *p *= 0.009 (adj.*p *= 0.11), being in an active control group vs. being in a MBSR or MBSR plus exercise, but not exercise alone group, was associated with poorer adherence) and race (*F*(3,210) = 3.86, *p *= 0.01 (adj.*p *= 0.06)), being White vs. Black or African American, but not Asian or more than one race, was associated with higher adherence) did not remain significant after correction for multiple testing. Adherence was not predicted by gender (*F*(1, 212) = 3.02, *p *= 0.08), age (*F*(1, 212) = 0.06, *p *= 0.80), ethnicity (*F*(1,212) = 0.36, *p *= 0.55), employment status (*F*(3,205) = 0.08, *p *= 0.97), residence type (*F*(2,206) = 1.1, *p *= 0.34), location (*F*(1,212) = 0.58, *p *= 0.45), generations in household (*F*(2,206) = 0.03, *p *= 0.97) or how often the house was left before the pandemic (*F*(3, 205) = 1.07, *p *= 0.36).

To follow up whether the trend-level association of lower adherence with people living with 2 or more people vs. living alone might be moderated by pandemic-related events, we examined whether increased care duties for family members or the presence of COVID-19 infections in the household might moderate this relationship.

However, in multivariate models, we found no relationship of adherence with a COVID-19 diagnosis of a family member (*F*(1,170) = 0.02, *p *= 0.89) and no interaction of the number of people in residence with COVID 19 infections of family members (*F*(2, 170) = 0.056, *p *= 0.95). Further, adherence was not related to whether the participant was giving support to a family member (*F*(1,160) = 0.42, *p *= 0.52) and there was no interaction effect of number of people in residence with giving support to a family member (*F*(2, 160) = 0.56, *p *= 0.57), indication no moderating effects of variables related to COVID19.

**Figure 2:**
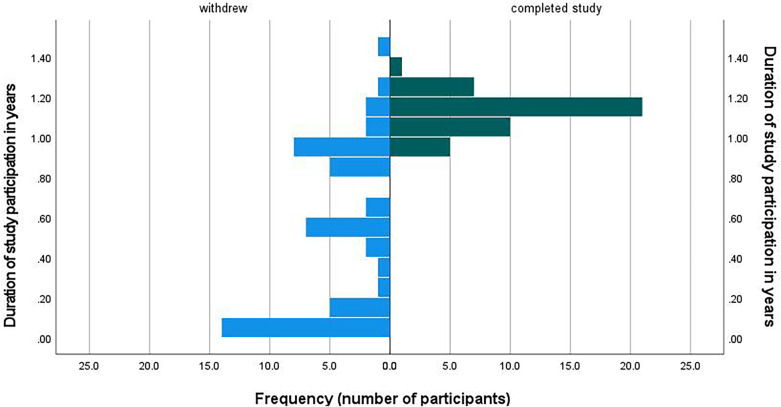
Distribution of study participation duration among participants who withdrew and who did not withdraw.

*Structural factors* related to adherence were duration of study participation (*F*(1, 212) = 11.7, *p *< 0.001, adj.*p *= 0.004) with a longer study participation associated with higher adherence, reaching burst 3 ((*F*(1, 212) = 9.84, *p *= 0.002 (adj.*p *= 0.054), with higher adherence in participants that made it to burst 3), and time elapsed between study start and enrollment (*F*(1,212) = 5.18, *p* = 0.02 (adj.*p *= 0.004), with a faster enrollment associated with a higher adherence). Structural factors not related to adherence were number of changes of RA's (*F*(1, 211) = 2.36, *p *= 0.09) and operating system (*F*(1,212) = 0.125, *p *= 0.26). When combining all significant predictors into one model, study participation duration and time between study start and enrollment remain significant predictors of adherence, while reaching burst 3 was not significant anymore (*F*(1, 210) = 2.97, *p *= 0.08), indicating its dependency on time elapsing (see Figure. 2 for a histogram of study participation duration in participants that withdrew and that did not withdraw).

### Adherence to EMA surveys does not differ between bursts

Within those participants (*n* = 42) that did not withdraw and that did contribute data to burst 1 and/or 2 and to burst 3 and/or 4 we found, contrary to our hypothesis, that adherence did not significantly differ between bursts 1/2 (81.7% ± 23.1) and bursts 3/4 (81.4% ± 20.3; *F*(1, 116) = 0.002, *p *= 0.965). When analyzing the four bursts separately, adherence during all four bursts was above 80% (see Table 4).

**Table 4:** Adherence rates across bursts in subsample of participants that contributed data to burst 1 and/or 2 and burst 3 and/or 4.

Completed EMA surveys	n (participants)	Adherence (mean %)	Standard deviation
burst1	42	82.6	22.7
burst2	40	80.8	23.8
burst3	38	82.8	18.6
burst4	38	80.1	22.0

## Conclusions and discussion

This longitudinal, observational study was the first of its kind, using daily smartphone EMA self-report surveys to investigate baseline and structural factors that predict study withdrawal and adherence in older adults across an extended time period covering multiple phases of the COVID 19 pandemic. The objective of this study was to assess factors that predict withdrawal and adherence to EMA in older adults over the course of a seventeen-month period, beginning during the sudden and long-term shutdown of normal behavior and routines and a forced shift to digital technology during the pandemic.

Our *main results* are that withdrawal was associated with less research staff changes and was less likely among participants who reached the study mid-point. No baseline characteristics predicted withdrawal. Main reasons for withdrawal were communication issues, i.e. staff not being able to contact participants.

We found an adherence rate of 82% and no fatigue effects. Adherence was predicted by education status, study participation duration, reaching the study midpoint and time between study start and enrollment. COVID infections or supporting people in the household was not related to adherence.

Our *first aim* was to explore reasons for *withdrawal* and if any personal baseline factors predicted withdrawal in older adults, including age, gender, race/ethnicity, employment, residence, number of people in residence, education, location, generations in the household, how often the house was left pre-pandemic and previous participation in an active treatment arm of the prior study randomized controlled trial (vs. a control condition). Further, we examined if study structural factors, including study research staff turnover, smartphone operating system, time elapsed between study start and enrollment and reaching the study mid-point predicted withdrawal. Based on the available literature in older adults, we hypothesized to find withdrawal due to participant burden (8) or communication issues (22).

Withdrawal was not predicted by baseline factors, but by number of changes of RA's and making it beyond the mid-study point. While it seems intuitive that people who complete the study reached the study mid-point, it is interesting that completing the study was associated with having 1 RA change,—and when combining significant structural factors into one model- with 2 RA changes compared to experiencing no change in RA. One potential explanation for this finding could be that participants who withdrew before/during the first burst did not experience any change in RA's, however when excluding participants who withdrew before or during the first burst, the results remain unchanged, indicating that this finding is not solely driven by having less opportunity to experience an RA change in the case of an earlier withdrawal. Further explanations could be that our findings suggest that once participants take part in the study for a certain amount of time (i.e. reached the study mid-point), they are likely to complete the study, potentially due to a sense of duty to finish what they started, even in the face of changes in personnel which was experienced more often the longer one was in the study. Furthermore, while changes in RA's depend on study participation duration, experiencing RA changes does not necessarily predict withdrawal. A possible interpretation could be that being assigned to a new RA led to more contact with study personnel and to different ways of explaining instructions on how to handle the study app, which might have been pleasing and helpful for participants, motivating them to stay in the study. Another explanation could be that participants did not want to withdraw upon a new contact in order to avoid making the newly assigned RA feel responsible for their withdrawal. While our study experienced a complex flow of personnel during the pandemic with many changes in the longer break in the middle of the study, our findings demonstrate that personnel change in itself is not predictive of withdrawal of participants and may actually help keep people in the study. We further demonstrate that the main reasons for withdrawal were communication issues, underlining the importance of forming reliable relationships with participants, that might even overcome technical issues, which we found to be only the fourth most frequent reason for withdrawal. Anecdotally, participants were very tolerant of technical issues if the situation was communicated transparently. This is in line with the literature emphasizing the importance of trust for the adaptation of digital technologies by older people (29) and with a study describing as a primary withdrawal reason that participants did not fully understand what they were supposed to do (22). Another withdrawal reason that was mentioned in one study was medical emergency (8), which we do not find in our study as a main withdrawal reason, possibly due to the very good initial health of our participants or a potential reduction of non-urgent medical treatments conducted during the pandemic.

These findings on withdrawal can be seen parallel to a previous finding on adherence, with early adherence predicting study-long adherence in adults (3). We further observed that adherence did not predict withdrawal, indicating that different factors might be at play in the two situations.

The *second aim* was to examine if any personal baseline or study structural factors predicted *adherence—*per burst—in older adults, based on the same personal and structural factors explored in relation to withdrawal. We hypothesized that adherence will be predicted by age and gender with highest adherence in younger women based on the literature on specific findings on older adults (21), but not by other personal factors (8, 19). Based on the literature, we further hypothesized that an Android operating system would be related to lower adherence (23).

Consistent with the EMA literature in older adults (8, 16, 19, 21), we found an adherence rate of 82% and no fatigue effects. Inconsistent with our hypothesis, we found no relationship between gender and age with adherence, which is line with a recent study in older people that reported no relation of age to completion or response rate, but found that older participants were more likely to report not being alerted to surveys (23): inconsistencies in findings might be due because adherence in older adults might be malleable by prompt support if participants contact research staff if they notice that their alerts do not come through as planned. Further, we found that more years of education were associated with higher adherence, potentially due to an increased exposure of our participants to research along their educational path and therefore potentially a sense of duty to contribute to research studies. There was a trend towards lower adherence among those living with two or more people compared to living alone. As a follow-up analysis, we hypothesized that people whose family member had a COVID-19 diagnosis or who were giving support to family members might be more likely to show such a negative relationship between the number of people living in the household and adherence rate. We observed no moderating effect of these two variables, and they also did not predict adherence themselves, adding novel evidence to the literature that EMA surveys can be a reliable tool during a pandemic in older adults and further that the adherence of older adults to EMA surveys was little impacted by the pandemic.

Regarding structural factors, the association of higher adherence with longer study participation and reaching the study mid-point is in line with the literature (3). Further we observed higher engagement in participants that enrolled faster in the study, potentially because participants that were highly motivated contacted the study staff faster, which is also in line with the literature (23). Additionally, we found no relationship of smartphone operating system with adherence, which is opposed to a previous finding of higher EMA response rate in iOS vs. Android users in a week-long study in older adults (23). This difference might be due to the difference in the duration of our study which lasted over several months vs. a 1-week long study, in such a way that a longer study duration allowed for more occasions to practice and get familiar with potential pitfalls of a specific operating system.

The *final aim* was to understand how the rate of completed EMA surveys from the first two bursts compared to the last two bursts. We hypothesized that adherence would be significantly less during burst 3 and 4 due to participant's reengaging in activities outside the home after spending more time at home during the pandemic (30).

Inconsistent with our hypothesis, interestingly, we did not find a difference in adherence between different time-points in the pandemic among those participants that did continue their study participation beyond the study mid-point– those who remained in the study were as adherent after lifting of restrictions as the larger group had been during early months of the pandemic, a finding that is to some extent in line with a previous finding that early adherence predicted study-long adherence in adults (3) and contrary to our initial hypothesis that more activities, as the stay-at-home restrictions lift, might lead to less adherence. This also adds weight to the interpretation that participants develop a loyalty towards the study, as their participation continues, potentially due to increasing trust and relationships with study personnel.

*Strengths* of this study include the extended time period covered during multiple phases of the pandemic and the availability of detailed pre-pandemic and during-pandemic baseline and structural variables.

*Limitations* of our study include the small sample size and bias of our sample: our sample has a high percentage of female participants and is not very diverse in terms of race, ethnicity and socioeconomic background. This is relevant especially because people from different backgrounds might have been affected differently by the pandemic. Further, all our participants owned their own smartphone, which might not be the case for socioeconomic environments different from the US, with rates of smartphone ownership among older adults below 35% in emerging economies (31), which further limits the generalizability of our results. Another factor is our study design that included a bonus payment of $20 for the completion of more than 85% of surveys, which was however not very high given the length of the study. Additionally, it is important to interpret the findings of our study in the light of relatively few surveys per day (2) and the possibility for scheduling a time window of 1 h for the random timing of the surveys, supporting a higher adherence. Further, it is a highly specific subgroup of participants who were concurrently enrolled in the MEDEX study that included some EMA components. However, the MEDEX EMA surveys were completed on study-provided tablets, while the StayWELL study used the participant's smartphone, making the present EMA experience different to some extent in regard to the technical aspects. Therefore, participants in our sample were familiar with completing EMA surveys and had previously demonstrated good adherence, since all the recruited participants could be considered previous study completers and currently active study participants of the MEDEX study. While we did not assess this question, it was previously found that the majority of experienced EMA participants preferred to use their own smartphone vs. study-provided devices (23), which might add, on top of the training effect of previous EMA study participation, a familiarity with handling of the participant's own phone. Whether these benefits outweigh the benefits of study-provided phones in preventing technical difficulties should be carefully considered in future studies, taking into consideration also costs of study-phones and the downside of having to manage two phones at once for the participants. In addition to familiarity with EMA procedures, the participants seemed to have found their previous study participation to be beneficial,, which seemed to motivate them to take part in the StayWELL study, which is in line with a previous finding, that previous study participation has led to higher enrollment and completion of an EMA study compared to inexperienced participants (23), potentially due to more contact/stronger relationships with research personnel and positive experiences. Furthermore, we did not follow up in detail about withdrawal reasons once withdrawal was communicated. Anecdotally, some of the detailed reasons related to communication issues were that some participants were difficult to reach for study staff *via* phone, due to e.g. not picking the phone up for non-identified caller IDs or due to being out/traveling. Reasons related to technical difficulties included running out of mobile data on the phone, too high battery drain or that the surveys did not come through to the phone. Based on individual, not systematically collected, more detailed feedback, it also became evident that for some participants it was not clear that their surveys would arrive at random times within the specific 1-hour time interval, and they were irritated because the surveys did not arrive on time with regular schedules. Further, the longer break between the second and third burst led some participants to believe that the study had ended without timely communication with them.

*Future* methodological questions arising are how to address missingness of data, whether to focus on subject withdrawal or to report adherence in the form of missing data per participant, since few outliers can have a large effect on the overall adherence rates (3, 16). Strategies for averaging data over the day or week have to be considered carefully, so information is not lost that might be valuable in predicting changes in adherence (2). Further, additional factors influencing adherence need to be considered: In EMA studies in healthy adults (18–65 years), higher anxiety and depression variables were correlated with lower adherence (3). A future outlook for our study is therefore to investigate the relationship of mood states with EMA adherence in older adults in order to contribute to recommendations how to best design EMA surveys that assess wellbeing in older adults (2). Additionally, an important next step will be to investigate whether these findings also apply to a diverse group of participants.

To *conclude*, EMA can potentially reach research participants unable to attend in-person study visits (e.g. due to restricted mobility) and allow for immediate collection with less bias. We hereby demonstrate that EMA surveys in older adults during unusual circumstances, such as a pandemic, to assess daily experiences are feasible and that engagement of older adults with EMA was high and little impacted by the phase of the pandemic. By investigating the factors underlying engagement and adherence to assess mental wellbeing using EMA in older adults in a situation of reduction of mobility, we identify barriers to consider when designing digital health technologies for research and clinical use in older people. We found that withdrawal and adherence are robust to study alterations and not associated with staff changes when accounting for study duration. This should be kept in mind when recruiting participants on the one hand, but also when building a cohort of participants and patients that might be willing to participate in several or longitudinal digital health studies or treatments on the other hand. We further demonstrate that communication issues were the largest contributing factor to withdrawal, suggesting that future digital health interventions should invest in an easy-to-use communication strategy for participants or patients that would like to contact their care provider or study personnel, a recommendation that also applies to study personnel, for which ease of use of digital platforms will also be a relevant factor in the success of a study. A further important finding is that the mobile software used by participants did not affect adherence or withdrawal, allowing for a broad distribution of digital health technologies. Our study adds information on single predictive variables that affect compliance to and withdrawal from EMA smartphone surveys in older people that can inform the design of future digital survey studies to maximize engagement and reliability of studies using EMA.

## Data Availability

The data that support the findings of this study are available on request from the corresponding author (FK) upon reasonable request and after filing an institutional data sharing agreement. The data are not publicly available due to the privacy of research participants. Requests to access the datasets should be directed to Federica Klaus, fklaus@health.ucsd.edu.
