# Supplementary material for: Mobile survey engagement by older adults is high during multiple phases of the COVID-19 pandemic and is predicted by baseline and structural factors
Source: Front Digit Health. 2022 Aug 23;4:920706. doi: 10.3389/fdgth.2022.920706 (PMC9445303; doi:10.3389/fdgth.2022.920706)
Supplement: Supplementary file 1 [file Table_1_v1.docx]

Supplementary Material

## Supplementary Data

**Supplementary Table S1** Demographics of complete set of participants and participants who completed burst 1 and/or 2 and burst 3 and/or 4.

|  | **All participants (n=95)** | | **Participants who completed burst 1 and/or 2 and burst 3 and/or 4 (n=42)** | |
| --- | --- | --- | --- | --- |
|  | **Mean (range) or n (%)** | **SD** | **Mean (range) or n (%)** | **SD** |
| **Age (years)** | 73.7 (67-87) | 4.3 | 73.4 (67-87) | 5.1 |
| **Gender (F (%))** | 74 (78%) | | 31 (74%) | |
| **Education (years)** | 16.6 | 2.0 | 16.8 | 1.5 |
| **Race (n (%))** |  | |  | |
| *White* | 80 (84%) | | 37 (88%) | |
| *Black/African American* | 8 (8%) | | 1 (2%) | |
| *Asian* | 4 (4%) | | 3 (7%) | |
| *More than one Race* | 3 (3%) | | 1 (2%) | |
| **Ethnicity (Non-Latino-Hispanic (%))** | 90 (95%) | | 41 (98%) | |
| **Employment status (Retired (%))** | 71 (90%) | | 38% (91%) | |
| **Number of people living in same household (0/1/2 or more)** | 28%/63%/9% | | 26%/64%/10% | |
| **Number of generations living in same household** | 0.3 | 0.5 | 0.4 | 0.6 |
| **How often house was left per day before pandemic (days per week)** | 4.2 | 0.9 | 4.0 | 0.9 |
| **COVID19 Diagnosis of family member (burst 1/2/3/4)** | 22%/19%/17%/22% | | 11%/9%/11%/15% | |
| **Support to family members given (burst 1/2/3/4)** | 34%/36%/36%/29% | | 30%/36%/26%/23% | |
| **Location (San Diego (%))** | 49 (52%) | | 23 (55%) | |
| **MEDEX intervention group** |  | |  | |
| *MBSR (mindfulness-based meditation)* | 27 (28%) | | 14 (33%) | |
| *Exercise* | 26 (27%) | | 10 (24%) | |
| *MBSR+Exercise* | 28 (29%) | | 11 (26%) | |
| *Health Education (comparison group)* | 14 (15%) | | 7 (17%) | |
| **Number of research assistant (RA) changes** | 0.5 | 0.7 | 0.6 | 0.6 |
| **Operating System (Android/iOS (%))** | 34 (36%)/61 (64%) | | 12 (29%)/30 (71%) | |
| **Bursts completed (0/1/2/3/4)** | 19/16/19/9/32 | | 0/0/1/10/31 | |
| **Last completed burst (0/1/2/3/4)** | 19/16/18/4/38 | | 0/0/0/4/38 | |
| **Withdrawal timing (until during burst 2/between burst 2 and 3/during or after burst 3)** | 27/19/49 | | 0/0/(42) | |
| **Study participation duration (years)** | 0.8 | 0.4 | 1.1 | 0.2 |
| **Time elapsed between study start and date of enrollment (months)** | 1.5 | 0.9 | 1.5 | 0.9 |

**Supplementary Table S2: Coefficients of Predictors of Withdrawal:** Target= Withdrawal

**Baseline factors**

| **Fixed Coefficients**^a^ | | | | | | | |
| --- | --- | --- | --- | --- | --- | --- | --- |
| Model Term | Coefficient | Std. Error | t | Sig. | 95% Confidence Interval | | Exp(Coefficient) |
|  |  |  |  |  | Lower | Upper |  |
| Intercept | -.486 | .4541 | -1.070 | .288 | -1.387 | .416 | .615 |
| Female | .648 | .5117 | 1.267 | .208 | -.368 | 1.664 | 1.912 |
| Male | 0^b^ | . | . | . | . | . | . |

| **Fixed Coefficients**^a^ | | | | | | |
| --- | --- | --- | --- | --- | --- | --- |
| Model Term | Coefficient | Std. Error | t | Sig. | 95% Confidence Interval | |
|  |  |  |  |  | Lower | Upper |
| Intercept | -.496 | 3.5151 | -.141 | .888 | -7.476 | 6.484 |
| Age (years) | .007 | .0476 | .147 | .883 | -.087 | .101 |

| **Fixed Coefficients**^a^ | | | | | | | |
| --- | --- | --- | --- | --- | --- | --- | --- |
| Model Term | Coefficient | Std. Error | t | Sig. | 95% Confidence Interval | | Exp(Coefficient) |
|  |  |  |  |  | Lower | Upper |  |
| Intercept | .433 | 1.7555 | .246 | .806 | -3.054 | 3.920 | 1.541 |
| Education (years) | -.025 | .1050 | -.236 | .814 | -.233 | .184 | .976 |

| **Fixed Coefficients**^a^ | | | | | | | |
| --- | --- | --- | --- | --- | --- | --- | --- |
| Model Term | Coefficient | Std. Error | t | Sig. | 95% Confidence Interval | | Exp(Coefficient) |
|  |  |  |  |  | Lower | Upper |  |
| Intercept | -.050 | .2284 | -.219 | .827 | -.504 | .404 | .951 |
| More than one race | .744 | 1.2685 | .587 | .559 | -1.776 | 3.264 | 2.105 |
| Asian | -1.050 | 1.1950 | -.878 | .382 | -3.423 | 1.324 | .350 |
| Black or African American | 1.150 | .8603 | 1.337 | .185 | -.559 | 2.859 | 3.158 |
| White | 0^b^ | . | . | . | . | . | . |

| **Fixed Coefficients**^a^ | | | | | | | | | | | | | | | | | | | | | |  |  |
| --- | --- | --- | --- | --- | --- | --- | --- | --- | --- | --- | --- | --- | --- | --- | --- | --- | --- | --- | --- | --- | --- | --- | --- |
| Model Term | Coefficient | | Std. Error | | | t | | | Sig. | | | 95% Confidence Interval | | | | | | Exp(Coefficient) | | | |  |  |
|  |  |  |  |  |  |  |  |  |  |  |  | Lower | | | Upper | | |  |  |  |  |  |  |
| Intercept | -.044 | | .2128 | | | -.209 | | | .835 | | | -.467 | | | .378 | | | .957 | | | |  |  |
| Not hispanic | 1.431 | | 1.1447 | | | 1.250 | | | .214 | | | -.842 | | | 3.704 | | | 4.183 | | | |  |  |
| Hispanic or Latino | 0^b^ | | . | | | . | | | . | | | . | | | . | | | . | | | |  |  |
| **Fixed Coefficients**^a^ | | | | | | | | | | | | | | | | | | | | | | | |
| Model Term | | | Coefficient | | | Std. Error | | t | | | Sig. | | | 95% Confidence Interval | | | | | | | Exp(Coefficient) | | |
|  |  |  |  |  |  |  |  |  |  |  |  |  |  | Lower | | | | Upper | | |  |  |  |
| Intercept | | | -.205 | | | .2904 | | -.705 | | | .482 | | | -.782 | | | | .372 | | | .815 | | |
| St. Louis | | | .467 | | | .4181 | | 1.118 | | | .266 | | | -.363 | | | | 1.298 | | | 1.596 | | |
| San Diego | | | 0^b^ | | | . | | . | | | . | | | . | | | | . | | | . | | |
| **Fixed Coefficients**^a^ | | | | | | | | | | | | | | | | | | | | | |  |  |
| Model Term | | | | | Coefficient | | | | Std. Error | | | t | | | Sig. | | 95% Confidence Interval | | | | |  |  |
|  |  |  |  |  |  |  |  |  |  |  |  |  |  |  |  |  | Lower | | | Upper | |  |  |
| Intercept | | | | | -8.566 | | | | 72.4636 | | | -.118 | | | .906 | | -152.890 | | | 135.758 | |  |  |
| House | | | | | 8.135 | | | | 72.4641 | | | .112 | | | .911 | | -136.190 | | | 152.459 | |  |  |
| Apartment/Condominium | | | | | 8.566 | | | | 72.4660 | | | .118 | | | .906 | | -135.763 | | | 152.894 | |  |  |
| Independent living facility | | | | | 0^b^ | | | | . | | | . | | | . | | . | | | . | |  |  |

| **Fixed Coefficients**^a^ | | | | | | |
| --- | --- | --- | --- | --- | --- | --- |
| Model Term | Coefficient | Std. Error | t | Sig. | 95% Confidence Interval | |
|  |  |  |  |  | Lower | Upper |
| Intercept | -2.776E-16 | .5476 | .000 | 1.000 | -1.088 | 1.088 |
| MBSR | -.375 | .6787 | -.553 | .582 | -1.724 | .973 |
| Exercise | -.154 | .6799 | -.227 | .821 | -1.505 | 1.196 |
| MBST+Exercise | .589 | .6801 | .866 | .389 | -.762 | 1.940 |
| Health Education | 0^b^ | . | . | . | . | . |

| **Fixed Coefficients**^a^ | | | | | | | | | | | | | | | | | | | |  |  |  |
| --- | --- | --- | --- | --- | --- | --- | --- | --- | --- | --- | --- | --- | --- | --- | --- | --- | --- | --- | --- | --- | --- | --- |
| Model Term | | | | Coefficient | | | Std. Error | | t | | | Sig. | | | 95% Confidence Interval | | | | |  |  |  |
|  |  |  |  |  |  |  |  |  |  |  |  |  |  |  | Lower | | | Upper | |  |  |  |
| Intercept | | | | -.183 | | | .4376 | | -.417 | | | .678 | | | -1.054 | | | .689 | |  |  |  |
| 2 or more people living in same household | | | | -.106 | | | .8946 | | -.118 | | | .906 | | | -1.887 | | | 1.676 | |  |  |  |
| 1 person living in same household | | | | -.308 | | | .5291 | | -.582 | | | .563 | | | -1.361 | | | .746 | |  |  |  |
| Living alone | | | | 0^b^ | | | . | | . | | | . | | | . | | | . | |  |  |  |
| **Fixed Coefficients**^a^ | | | | | | | | | | | | | | | | | | | | | | |
| Model Term | | Coefficient | Std. Error | | | t | | | Sig. | | | 95% Confidence Interval | | | | | | Exp(Coefficient) | | | | |
|  |  |  |  |  |  |  |  |  |  |  |  | Lower | | | Upper | | |  |  |  |  |  |
| Intercept | | -.205 | .2904 | | | -.705 | | | .482 | | | -.782 | | | .372 | | | .815 | | | | |
| St. Louis | | .467 | .4181 | | | 1.118 | | | .266 | | | -.363 | | | 1.298 | | | 1.596 | | | | |
| San Diego | | 0^b^ | . | | | . | | | . | | | . | | | . | | | . | | | | |
| **Fixed Coefficients**^a^ | | | | | | | | | | | | | | | | | | | | | |  |
| Model Term | | | | Coefficient | | | Std. Error | | | | t | | | Sig. | | | 95% Confidence Interval | | | | |  |
|  |  |  |  |  |  |  |  |  |  |  |  |  |  |  |  |  | Lower | | | Upper | |  |
| Intercept | | | | -.278 | | | .2689 | | | | -1.033 | | | .305 | | | -.813 | | | .258 | |  |
| 2 Generations living in household (excl self) | | | | -17.288 | | | 3765.8472 | | | | -.005 | | | .996 | | | -7517.623 | | | 7483.046 | |  |
| 1 Generation living in household (excl self) | | | | -.174 | | | .5590 | | | | -.312 | | | .756 | | | -1.288 | | | .939 | |  |
| 0 Generations living in household (excl self) | | | | 0^b^ | | | . | | | | . | | | . | | | . | | | . | |  |

| **Fixed Coefficients**^a^ | | | | | | | | | | | | |  |  |
| --- | --- | --- | --- | --- | --- | --- | --- | --- | --- | --- | --- | --- | --- | --- |
| Model Term | | Coefficient | | Std. Error | t | | Sig. | | 95% Confidence Interval | | | |  |  |
|  |  |  |  |  |  |  |  |  | Lower | | Upper | |  |  |
| Intercept | | 1.110E-16 | | 1.0307 | .000 | | 1.000 | | -2.053 | | 2.053 | |  |  |
| House was left before pandemic every day | | -1.110E-16 | | 1.0836 | .000 | | 1.000 | | -2.159 | | 2.159 | |  |  |
| House was left before pandemic 5-6 days/week | | -.621 | | 1.1378 | -.546 | | .587 | | -2.887 | | 1.646 | |  |  |
| House was left before pandemic 3-4 days/week | | -1.181 | | 1.1848 | -.997 | | .322 | | -3.541 | | 1.179 | |  |  |
| House was left before pandemic 1-2 days/week | | 0^b^ | | . | . | | . | | . | | . | |  |  |
| **Fixed Coefficients**^a^ | | | | | | | | | | | | | | |
| Model Term | | Coefficient | | Std. Error | | | t | | Sig. | | 95% Confidence Interval | | | |
|  |  |  |  |  |  |  |  |  |  |  | Lower | | Upper | |
| Intercept | | -.694 | | 1.2489 | | | -.556 | | .580 | | -3.182 | | 1.794 | |
| Homemaker | | 17.260 | | 2797.4422 | | | .006 | | .995 | | -5555.530 | | 5590.051 | |
| Retired | | .252 | | 1.2741 | | | .197 | | .844 | | -2.286 | | 2.790 | |
| Working for pay at home | | .288 | | 1.5586 | | | .185 | | .854 | | -2.817 | | 3.393 | |
| Working for pay outside the house | | 0^b^ | | . | | | . | | . | | . | | . | |

**Structural factors**

| **Fixed Coefficients**^a^ | | | | | | | |
| --- | --- | --- | --- | --- | --- | --- | --- |
| Model Term | Coefficient | Std. Error | t | Sig. | 95% Confidence Interval | | Exp(Coefficient) |
|  |  |  |  |  | Lower | Upper |  |
| Intercept | .669 | .2800 | 2.388 | .019 | .113 | 1.225 | 1.952 |
| 2 changes of RA | -1.180 | .7950 | -1.485 | .141 | -2.759 | .399 | .307 |
| 1 change of RA | -1.969 | .5443 | -3.618 | <.001 | -3.050 | -.888 | .140 |
| No changes of RA | 0^b^ | . | . | . | . | . | . |

| **Fixed Coefficients**^a^ | | | | | | | | | | | | | | | | | | | | |  |  |
| --- | --- | --- | --- | --- | --- | --- | --- | --- | --- | --- | --- | --- | --- | --- | --- | --- | --- | --- | --- | --- | --- | --- |
| Model Term | Coefficient | | | | Std. Error | | | t | | | Sig. | | | 95% Confidence Interval | | | | | | Exp(Coefficient) |  |  |
|  |  |  |  |  |  |  |  |  |  |  |  |  |  | Lower | | | Upper | | |  |  |  |
| Intercept | 2.047 | | | | .4316 | | | 4.743 | | | <.001 | | | 1.190 | | | 2.904 | | | 7.746 |  |  |
| Reached study mid-point | -5.171 | | | | .8809 | | | -5.869 | | | <.001 | | | -6.920 | | | -3.421 | | | .006 |  |  |
| Did not reach study mid-point | 0^b^ | | | | . | | | . | | | . | | | . | | | . | | | . |  |  |
| **Fixed Coefficients**^a^ | | | | | | | | | | | | | | | | | | | | | | |
| Model Term | | | | Coefficient | | | | Std. Error | | | t | | | Sig. | | | 95% Confidence Interval | | | | | |
|  |  |  |  |  |  |  |  |  |  |  |  |  |  |  |  |  | Lower | | | Upper | | |
| Intercept | | | | 4.281 | | | | 1.0920 | | | 3.921 | | | <.001 | | | 2.113 | | | 6.450 | | |
| Duration of study participation | | | | -4.902 | | | | 1.0936 | | | -4.482 | | | <.001 | | | -7.074 | | | -2.730 | | |
| **Fixed Coefficients**^a^ | | | | | | | | | | | | | | | | | | | | | | |
| Model Term | | | Coefficient | | Std. Error | | t | | | Sig. | | | 95% Confidence Interval | | | | | | Exp(Coefficient) | | | |
|  |  |  |  |  |  |  |  |  |  |  |  |  | Lower | | | Upper | | |  |  |  |  |
| Intercept | | | -.075 | | .4181 | | -.179 | | | .858 | | | -.905 | | | .755 | | | .928 | | | |
| Time between study start and participant enrollment | | | .064 | | .2414 | | .264 | | | .792 | | | -.416 | | | .543 | | | 1.066 | | | |

| **Fixed Coefficients^a^** | | | | | | | | | |
| --- | --- | --- | --- | --- | --- | --- | --- | --- | --- |
| Model Term | Coefficient | Std. Error | t | Sig. | 95% Confidence Interval | | Exp(Coefficient) | 95% Confidence Interval for Exp(Coefficient) | |
|  |  |  |  |  | Lower | Upper |  | Lower | Upper |
| Intercept | .118 | .3474 | .339 | .735 | -.572 | .808 | 1.125 | .564 | 2.243 |
| iOS | -.151 | .4333 | -.348 | .729 | -1.011 | .710 | .860 | .364 | 2.034 |
| Android | 0^b^ | . | . | . | . | . | . | . | . |

| **Fixed Coefficients**^a^ | | | | | | | |
| --- | --- | --- | --- | --- | --- | --- | --- |
| Model Term | Coefficient | Std. Error | t | Sig. | 95% Confidence Interval | | Exp(Coefficient) |
|  |  |  |  |  | Lower | Upper |  |
| Intercept | 2.820 | .6597 | 4.275 | <.001 | 1.510 | 4.130 | 16.776 |
| Reached study mid-point | -5.147 | .9233 | -5.575 | <.001 | -6.981 | -3.313 | .006 |
| Did not reach study mid-point | 0^b^ | . | . | . | . | . | . |
| 2 changes of RA | -2.390 | 1.1131 | -2.147 | .034 | -4.601 | -.179 | .092 |
| 1 change of RA | -1.881 | .9108 | -2.065 | .042 | -3.690 | -.071 | .153 |
| No changes of RA | 0^b^ | . | . | . | . | . | . |

| **Fixed Coefficients**^a^ | | | | | | |
| --- | --- | --- | --- | --- | --- | --- |
| Model Term | Coefficient | Std. Error | t | Sig. | 95% Confidence Interval | |
|  |  |  |  |  | Lower | Upper |
| Intercept | 4.233 | 1.0908 | 3.880 | <.001 | 2.066 | 6.400 |
| Reached study mid-point | -4.393 | .9221 | -4.764 | <.001 | -6.225 | -2.561 |
| Did not reach study mid-point | 0^b^ | . | . | . | . | . |
| Duration of study participation | -2.303 | 1.0897 | -2.113 | .037 | -4.468 | -.138 |
| 2 changes of RA | -1.611 | 1.1429 | -1.410 | .162 | -3.882 | .659 |
| 1 change of RA | -1.349 | .9133 | -1.477 | .143 | -3.163 | .466 |
| No changes of RA | 0^b^ | . | . | . | . | . |

**Adherence as predictor of withdrawal**

| **Fixed Coefficients**^a^ | | | | | | |
| --- | --- | --- | --- | --- | --- | --- |
| Model Term | Coefficient | Std. Error | t | Sig. | 95% Confidence Interval | |
|  |  |  |  |  | Lower | Upper |
| Intercept | .916 | .7827 | 1.170 | .246 | -.645 | 2.476 |
| Adherence | -.019 | .0101 | -1.894 | .062 | -.039 | .001 |

**Supplementary Table S3: Coefficients of Predictors of Adherence**

**Baseline factors**

| **Fixed Coefficients**^a^ | | | | | | |
| --- | --- | --- | --- | --- | --- | --- |
| Model Term | Coefficient | Std. Error | t | Sig. | 95% Confidence Interval | |
|  |  |  |  |  | Lower | Upper |
| Intercept | 59.241 | 7.8297 | 7.566 | <.001 | 43.806 | 74.676 |
| burst=1 | 4.705 | 3.9135 | 1.202 | .231 | -3.010 | 12.419 |
| burst=2 | -6.976 | 3.9628 | -1.760 | .080 | -14.788 | .836 |
| burst=3 | 2.646 | 4.3287 | .611 | .542 | -5.888 | 11.179 |
| burst=4 | 0^b^ | . | . | . | . | . |
| MBSR | 20.180 | 8.7477 | 2.307 | .022 | 2.935 | 37.425 |
| Exercise | 8.873 | 8.9116 | .996 | .321 | -8.695 | 26.440 |
| MBST+Exercise | 25.784 | 8.7282 | 2.954 | .003 | 8.578 | 42.990 |
| Health Education | 0^b^ | . | . | . | . | . |
| Probability distribution: Normal Link function: Identity ^a^ | | | | | | |
| a. Target: Adherence | | | | | | |
| b. This coefficient is set to zero because it is redundant. | | | | | | |

| **Fixed Coefficients**^a^ | | | | | | |
| --- | --- | --- | --- | --- | --- | --- |
| Model Term | Coefficient | Std. Error | t | Sig. | 95% Confidence Interval | |
|  |  |  |  |  | Lower | Upper |
| Intercept | 71.642 | 5.9614 | 12.018 | .000 | 59.890 | 83.393 |
| burst=4 | -5.105 | 3.8936 | -1.311 | .191 | -12.780 | 2.570 |
| burst=3 | -2.390 | 3.8941 | -.614 | .540 | -10.066 | 5.286 |
| burst=2 | -11.751 | 3.1529 | -3.727 | <.001 | -17.966 | -5.536 |
| burst=1 | 0^b^ | . | . | . | . | . |
| Female | 11.315 | 6.5131 | 1.737 | .084 | -1.524 | 24.154 |
| Male | 0^b^ | . | . | . | . | . |
| Probability distribution: Normal Link function: Identity ^a^ | | | | | | |
| a. Target: Adherence | | | | | | |
| b. This coefficient is set to zero because it is redundant. | | | | | | |

| **Fixed Coefficients**^a^ | | | | | | |
| --- | --- | --- | --- | --- | --- | --- |
| Model Term | Coefficient | Std. Error | t | Sig. | 95% Confidence Interval | |
|  |  |  |  |  | Lower | Upper |
| Intercept | 68.670 | 47.1678 | 1.456 | .147 | -24.308 | 161.648 |
| burst=4 | -5.327 | 3.8976 | -1.367 | .173 | -13.010 | 2.356 |
| burst=3 | -2.618 | 3.8979 | -.672 | .503 | -10.301 | 5.066 |
| burst=2 | -11.685 | 3.1548 | -3.704 | <.001 | -17.904 | -5.466 |
| burst=1 | 0^b^ | . | . | . | . | . |
| Age (years) | .159 | .6400 | .248 | .804 | -1.103 | 1.420 |
| Probability distribution: Normal Link function: Identity ^a^ | | | | | | |
| a. Target: Adherence | | | | | | |
| b. This coefficient is set to zero because it is redundant. | | | | | | |

| **Fixed Coefficients**^a^ | | | | | | | | | | | | | |
| --- | --- | --- | --- | --- | --- | --- | --- | --- | --- | --- | --- | --- | --- |
| Model Term | | Coefficient | | Std. Error | | t | | Sig. | | 95% Confidence Interval | | | |
|  |  |  |  |  |  |  |  |  |  | Lower | | Upper | |
| Intercept | | 21.806 | | 23.5536 | | .926 | | .356 | | -24.631 | | 68.243 | |
| burst=4 | | -5.320 | | 3.9825 | | -1.336 | | .183 | | -13.172 | | 2.532 | |
| burst=3 | | -3.152 | | 4.0242 | | -.783 | | .434 | | -11.086 | | 4.782 | |
| burst=2 | | -11.984 | | 3.2138 | | -3.729 | | <.001 | | -18.320 | | -5.648 | |
| burst=1 | | 0^b^ | | . | | . | | . | | . | | . | |
| Education (years) | | 3.522 | | 1.4069 | | 2.503 | | .013 | | .748 | | 6.295 | |
| Probability distribution: Normal Link function: Identity ^a^ | | | | | | | | | | | | | |
| a. Target: Adherence | | | | | | | | | | | | | |
| b. This coefficient is set to zero because it is redundant. | | | | | | | | | | | | | |
| Fixed Coefficientsa | | | | | | | | | | | | | |
| Model Term | | Coefficient | | Std. Error | | t | | Sig. | | 95% Confidence Interval | | | |
|  |  |  |  |  |  |  |  |  |  | Lower | | Upper | |
| Intercept | | 81.094 | | 3.3095 | | 24.504 | | .000 | | 74.570 | | 87.618 | |
| burst=4 | | -5.571 | | 3.8982 | | -1.429 | | .154 | | -13.256 | | 2.114 | |
| burst=3 | | -2.771 | | 3.8967 | | -.711 | | .478 | | -10.453 | | 4.911 | |
| burst=2 | | -11.805 | | 3.1566 | | -3.740 | | <.001 | | -18.027 | | -5.582 | |
| burst=1 | | 0^b^ | | . | | . | | . | | . | | . | |
| More than one race | | 19.835 | | 13.4300 | | 1.477 | | .141 | | -6.640 | | 46.310 | |
| Asian | | 12.644 | | 12.8687 | | .983 | | .327 | | -12.724 | | 38.012 | |
| Black or African American | | -30.399 | | 10.9180 | | -2.784 | | .006 | | -51.922 | | -8.876 | |
| White | | 0^b^ | | . | | . | | . | | . | | . | |
| Probability distribution: Normal Link function: Identity ^a^ | | | | | | | | | | | | | |
| a. Target: Adherence | | | | | | | | | | | | | |
| b. This coefficient is set to zero because it is redundant. | | | | | | | | | | | | | |

| **Fixed Coefficients**^a^ | | | | | | |
| --- | --- | --- | --- | --- | --- | --- |
| Model Term | Coefficient | Std. Error | t | Sig. | 95% Confidence Interval | |
|  |  |  |  |  | Lower | Upper |
| Intercept | 80.813 | 3.3498 | 24.124 | .000 | 74.210 | 87.416 |
| burst=4 | -5.404 | 3.8988 | -1.386 | .167 | -13.089 | 2.281 |
| burst=3 | -2.686 | 3.8994 | -.689 | .492 | -10.373 | 5.000 |
| burst=2 | -11.664 | 3.1547 | -3.697 | <.001 | -17.883 | -5.446 |
| burst=1 | 0^b^ | . | . | . | . | . |
| Not hispanic | -6.746 | 11.3170 | -.596 | .552 | -29.055 | 15.562 |
| Hispanic or Latino | 0^b^ | . | . | . | . | . |
| Probability distribution: Normal Link function: Identity ^a^ | | | | | | |
| a. Target: Adherence | | | | | | |
| b. This coefficient is set to zero because it is redundant. | | | | | | |

| **Fixed Coefficients**^a^ | | | | | | |
| --- | --- | --- | --- | --- | --- | --- |
| Model Term | Coefficient | Std. Error | t | Sig. | 95% Confidence Interval | |
|  |  |  |  |  | Lower | Upper |
| Intercept | 78.397 | 4.1468 | 18.905 | .000 | 70.222 | 86.571 |
| burst=4 | -5.332 | 3.8978 | -1.368 | .173 | -13.016 | 2.351 |
| burst=3 | -2.632 | 3.8986 | -.675 | .500 | -10.317 | 5.052 |
| burst=2 | -11.703 | 3.1557 | -3.709 | <.001 | -17.924 | -5.483 |
| burst=1 | 0^b^ | . | . | . | . | . |
| St. Louis | 4.267 | 5.5883 | .764 | .446 | -6.748 | 15.283 |
| San Diego | 0^b^ | . | . | . | . | . |
| Probability distribution: Normal Link function: Identity ^a^ | | | | | | |
| a. Target: Adherence | | | | | | |
| b. This coefficient is set to zero because it is redundant. | | | | | | |

| **Fixed Coefficients**^a^ | | | | | | |
| --- | --- | --- | --- | --- | --- | --- |
| Model Term | Coefficient | Std. Error | t | Sig. | 95% Confidence Interval | |
|  |  |  |  |  | Lower | Upper |
| Intercept | 97.454 | 23.2635 | 4.189 | <.001 | 51.589 | 143.319 |
| burst=4 | -5.134 | 3.8333 | -1.339 | .182 | -12.692 | 2.423 |
| burst=3 | -2.493 | 3.8335 | -.650 | .516 | -10.050 | 5.065 |
| burst=2 | -10.759 | 3.1400 | -3.426 | <.001 | -16.950 | -4.568 |
| burst=1 | 0^b^ | . | . | . | . | . |
| House | -18.874 | 23.3866 | -.807 | .421 | -64.982 | 27.234 |
| Apartment/Condominium | -8.628 | 24.3102 | -.355 | .723 | -56.557 | 39.300 |
| Independent living facility | 0^b^ | . | . | . | . | . |
| Probability distribution: Normal Link function: Identity ^a^ | | | | | | |
| a. Target: Adherence | | | | | | |
| b. This coefficient is set to zero because it is redundant. | | | | | | |

| **Fixed Coefficients**^a^ | | | | | | |
| --- | --- | --- | --- | --- | --- | --- |
| Model Term | Coefficient | Std. Error | t | Sig. | 95% Confidence Interval | |
|  |  |  |  |  | Lower | Upper |
| Intercept | 89.206 | 5.3576 | 16.650 | .000 | 78.639 | 99.773 |
| burst=4 | -5.465 | 4.0728 | -1.342 | .181 | -13.499 | 2.568 |
| burst=3 | -3.628 | 4.1936 | -.865 | .388 | -11.899 | 4.644 |
| burst=2 | -10.806 | 3.1032 | -3.482 | <.001 | -16.926 | -4.685 |
| burst=1 | 0^b^ | . | . | . | . | . |
| 2 or more people living in same household | -24.900 | 8.3244 | -2.991 | .003 | -41.319 | -8.481 |
| 1 person living in same household | -8.485 | 5.5506 | -1.529 | .128 | -19.433 | 2.463 |
| Living alone | 0^b^ | . | . | . | . | . |
| Probability distribution: Normal Link function: Identity ^a^ | | | | | | |
| a. Target: Adherence | | | | | | |
| b. This coefficient is set to zero because it is redundant. | | | | | | |

| **Fixed Coefficients**^a^ | | | | | | |
| --- | --- | --- | --- | --- | --- | --- |
| Model Term | Coefficient | Std. Error | t | Sig. | 95% Confidence Interval | |
|  |  |  |  |  | Lower | Upper |
| Intercept | 79.975 | 3.8390 | 20.832 | .000 | 72.406 | 87.544 |
| burst=4 | -5.215 | 3.8389 | -1.359 | .176 | -12.784 | 2.353 |
| burst=3 | -2.494 | 3.8387 | -.650 | .517 | -10.063 | 5.074 |
| burst=2 | -10.863 | 3.1417 | -3.458 | <.001 | -17.057 | -4.669 |
| burst=1 | 0^b^ | . | . | . | . | . |
| 2 Generations living in household (excl self) | .859 | 14.0388 | .061 | .951 | -26.819 | 28.537 |
| 1 Generation living in household (excl self) | 1.662 | 6.6750 | .249 | .804 | -11.499 | 14.822 |
| 0 Generations living in household (excl self) | 0^b^ | . | . | . | . | . |
| Probability distribution: Normal Link function: Identity ^a^ | | | | | | |
| a. Target: Adherence | | | | | | |
| b. This coefficient is set to zero because it is redundant. | | | | | | |

| **Fixed Coefficients**^a^ | | | | | | |
| --- | --- | --- | --- | --- | --- | --- |
| Model Term | Coefficient | Std. Error | t | Sig. | 95% Confidence Interval | |
|  |  |  |  |  | Lower | Upper |
| Intercept | 67.055 | 14.0646 | 4.768 | <.001 | 39.325 | 94.785 |
| burst=4 | -5.442 | 3.8418 | -1.417 | .158 | -13.017 | 2.132 |
| burst=3 | -2.713 | 3.8417 | -.706 | .481 | -10.287 | 4.862 |
| burst=2 | -10.817 | 3.1438 | -3.441 | <.001 | -17.015 | -4.619 |
| burst=1 | 0^b^ | . | . | . | . | . |
| House was left before pandemic every day | 9.778 | 14.5894 | .670 | .503 | -18.987 | 38.542 |
| House was left before pandemic 5-6 days/week | 15.819 | 15.0689 | 1.050 | .295 | -13.891 | 45.529 |
| House was left before pandemic 3-4 days/week | 20.141 | 15.1098 | 1.333 | .184 | -9.650 | 49.931 |
| House was left before pandemic 1-2 days/week | 0^b^ | . | . | . | . | . |
| Probability distribution: Normal Link function: Identity ^a^ | | | | | | |
| a. Target: Adherence | | | | | | |
| b. This coefficient is set to zero because it is redundant. | | | | | | |

| **Fixed Coefficients**^a^ | | | | | | |
| --- | --- | --- | --- | --- | --- | --- |
| Model Term | Coefficient | Std. Error | t | Sig. | 95% Confidence Interval | |
|  |  |  |  |  | Lower | Upper |
| Intercept | 82.205 | 14.4982 | 5.670 | <.001 | 53.620 | 110.790 |
| burst=4 | -5.145 | 3.8408 | -1.340 | .182 | -12.718 | 2.428 |
| burst=3 | -2.433 | 3.8413 | -.633 | .527 | -10.007 | 5.140 |
| burst=2 | -10.844 | 3.1438 | -3.449 | <.001 | -17.042 | -4.645 |
| burst=1 | 0^b^ | . | . | . | . | . |
| Homemaker | 7.145 | 29.1719 | .245 | .807 | -50.370 | 64.661 |
| Retired | -1.829 | 14.7376 | -.124 | .901 | -30.885 | 27.228 |
| Working for pay at home | -7.064 | 22.0917 | -.320 | .749 | -50.620 | 36.492 |
| Working for pay outside the house | 0^b^ | . | . | . | . | . |
| Probability distribution: Normal Link function: Identity ^a^ | | | | | | |
| a. Target: Adherence | | | | | | |
| b. This coefficient is set to zero because it is redundant. | | | | | | |

| **Fixed Coefficients**^a^ | | | | | | |
| --- | --- | --- | --- | --- | --- | --- |
| Model Term | Coefficient | Std. Error | t | Sig. | 95% Confidence Interval | |
|  |  |  |  |  | Lower | Upper |
| Intercept | 89.643 | 4.4314 | 20.229 | .000 | 80.895 | 98.391 |
| burst=4 | -4.125 | 3.2970 | -1.251 | .213 | -10.633 | 2.384 |
| burst=3 | -2.839 | 3.3528 | -.847 | .398 | -9.457 | 3.780 |
| burst=2 | -4.919 | 2.6264 | -1.873 | .063 | -10.104 | .265 |
| burst=1 | 0^b^ | . | . | . | . | . |
| Family member had diagnosis of COVID | 1.736 | 6.5311 | .266 | .791 | -11.156 | 14.629 |
| No family member had diagnosis of COVID | 0^b^ | . | . | . | . | . |
| 2 or more people living in same household | -19.218 | 7.3498 | -2.615 | .010 | -33.727 | -4.709 |
| 1 person living in same household | -4.618 | 4.5953 | -1.005 | .316 | -13.689 | 4.454 |
| Living alone | 0^b^ | . | . | . | . | . |
| [Family member had diagnosis of COVID]*[2 or more people living in same household] | -1.216 | 10.3361 | -.118 | .907 | -21.619 | 19.188 |
| [Family member had diagnosis of COVID]*[1 person living in same household] | -2.581 | 7.8586 | -.328 | .743 | -18.094 | 12.932 |
| [Family member had diagnosis of COVID]*[Living alone] | 0^b^ | . | . | . | . | . |
| [No family member had diagnosis of COVID]*[2 or more people living in same household] | 0^b^ | . | . | . | . | . |
| [No family member had diagnosis of COVID]*[1 person living in same household] | 0^b^ | . | . | . | . | . |
| [No family member had diagnosis of COVID]*[Living alone] | 0^b^ | . | . | . | . | . |
| Probability distribution: Normal Link function: Identity ^a^ | | | | | | |
| a. Target: Adherence | | | | | | |
| b. This coefficient is set to zero because it is redundant. | | | | | | |

| **Fixed Coefficients**^a^ | | | | | | |
| --- | --- | --- | --- | --- | --- | --- |
| Model Term | Coefficient | Std. Error | t | Sig. | 95% Confidence Interval | |
|  |  |  |  |  | Lower | Upper |
| Intercept | 96.698 | 8.7487 | 11.053 | .000 | 79.420 | 113.976 |
| burst=4 | -2.204 | 3.2171 | -.685 | .494 | -8.558 | 4.149 |
| burst=3 | -2.770 | 3.2104 | -.863 | .390 | -9.110 | 3.570 |
| burst=2 | -4.002 | 2.5550 | -1.566 | .119 | -9.048 | 1.044 |
| burst=1 | 0^b^ | . | . | . | . | . |
| Not supporting family members | -7.029 | 8.0843 | -.870 | .386 | -22.995 | 8.936 |
| Supporting family members | 0^b^ | . | . | . | . | . |
| 2 or more people living in same household | -29.515 | 10.6368 | -2.775 | .006 | -50.522 | -8.509 |
| 1 person living in same household | -9.740 | 9.0022 | -1.082 | .281 | -27.519 | 8.038 |
| Living alone | 0^b^ | . | . | . | . | . |
| [Not supporting family members]*[2 or more people living in same household] | 10.974 | 11.7780 | .932 | .353 | -12.286 | 34.235 |
| [Not supporting family members]*[1 person living in same household] | 1.857 | 8.9327 | .208 | .836 | -15.784 | 19.498 |
| [Not supporting family members]*[Living alone] | 0^b^ | . | . | . | . | . |
| [Supporting family members]*[2 or more people living in same household] | 0^b^ | . | . | . | . | . |
| [Supporting family members]*[1 person living in same household] | 0^b^ | . | . | . | . | . |
| [Supporting family members]*[Living alone] | 0^b^ | . | . | . | . | . |
| Probability distribution: Normal Link function: Identity ^a^ | | | | | | |
| a. Target: Adherence | | | | | | |
| b. This coefficient is set to zero because it is redundant. | | | | | | |

**Structural factors**

| **Fixed Coefficients**^a^ | | | | | | |
| --- | --- | --- | --- | --- | --- | --- |
| Model Term | Coefficient | Std. Error | t | Sig. | 95% Confidence Interval | |
|  |  |  |  |  | Lower | Upper |
| Intercept | 75.234 | 4.1159 | 18.279 | .000 | 67.120 | 83.347 |
| burst=4 | -5.304 | 3.8979 | -1.361 | .175 | -12.988 | 2.380 |
| burst=3 | -2.469 | 3.8972 | -.634 | .527 | -10.151 | 5.213 |
| burst=2 | -11.601 | 3.1561 | -3.676 | <.001 | -17.822 | -5.379 |
| burst=1 | 0^b^ | . | . | . | . | . |
| 2 changes of RA | 18.088 | 9.7475 | 1.856 | .065 | -1.127 | 37.303 |
| 1 change of RA | 9.018 | 5.7945 | 1.556 | .121 | -2.404 | 20.441 |
| No changes of RA | 0^b^ | . | . | . | . | . |
| Probability distribution: Normal Link function: Identity ^a^ | | | | | | |
| a. Target: Adherence | | | | | | |
| b. This coefficient is set to zero because it is redundant. | | | | | | |

| **Fixed Coefficients**^a^ | | | | | | |
| --- | --- | --- | --- | --- | --- | --- |
| Model Term | Coefficient | Std. Error | t | Sig. | 95% Confidence Interval | |
|  |  |  |  |  | Lower | Upper |
| Intercept | 70.403 | 4.4541 | 15.807 | .000 | 61.624 | 79.183 |
| burst=4 | -7.545 | 3.9570 | -1.907 | .058 | -15.345 | .256 |
| burst=3 | -4.843 | 3.9588 | -1.223 | .223 | -12.647 | 2.961 |
| burst=2 | -11.891 | 3.1462 | -3.780 | <.001 | -18.093 | -5.689 |
| burst=1 | 0^b^ | . | . | . | . | . |
| Reached study mid-point | 17.538 | 5.5906 | 3.137 | .002 | 6.517 | 28.558 |
| Did not reach study mid-point | 0^b^ | . | . | . | . | . |
| Probability distribution: Normal Link function: Identity ^a^ | | | | | | |
| a. Target: Adherence | | | | | | |
| b. This coefficient is set to zero because it is redundant. | | | | | | |

| **Fixed Coefficients**^a^ | | | | | | | | | | | | | |
| --- | --- | --- | --- | --- | --- | --- | --- | --- | --- | --- | --- | --- | --- |
| Model Term | | | Coefficient | | Std. Error | | t | | Sig. | | 95% Confidence Interval | | |
|  |  |  |  |  |  |  |  |  |  |  | Lower | | Upper |
| Intercept | | | 54.403 | | 8.2177 | | 6.620 | | <.001 | | 38.204 | | 70.602 |
| burst=4 | | | -6.823 | | 3.9061 | | -1.747 | | .082 | | -14.523 | | .877 |
| burst=3 | | | -3.745 | | 3.8942 | | -.962 | | .337 | | -11.422 | | 3.931 |
| burst=2 | | | -12.142 | | 3.1435 | | -3.863 | | <.001 | | -18.339 | | -5.946 |
| burst=1 | | | 0^b^ | | . | | . | | . | | . | | . |
| Duration of study participation | | | 27.964 | | 8.1972 | | 3.411 | | <.001 | | 11.805 | | 44.122 |
| Probability distribution: Normal Link function: Identity ^a^ | | | | | | | | | | | | | |
| a. Target: Adherence | | | | | | | | | | | | | |
| b. This coefficient is set to zero because it is redundant. | | | | | | | | | | | | | |
| **Fixed Coefficients**^a^ | | | | | | | | | | | | | |
| Model Term | | Coefficient | Std. Error | | t | | Sig. | | 95% Confidence Interval | | | |  |
|  |  |  |  |  |  |  |  |  |  | | | |  |
|  |  |  |  |  |  |  |  |  | Lower | | Upper | |  |
| Intercept | | 91.764 | 5.9375 | | 15.455 | | .000 | | 80.060 | | 103.468 | |  |
| burst=4 | | -5.318 | 3.8910 | | -1.367 | | .173 | | -12.988 | | 2.351 | |  |
| burst=3 | | -2.833 | 3.8943 | | -.728 | | .468 | | -10.510 | | 4.843 | |  |
| burst=2 | | -11.880 | 3.1538 | | -3.767 | | <.001 | | -18.097 | | -5.663 | |  |
| burst=1 | | 0^b^ | . | | . | | . | | . | | . | |  |
| Time between study start and participant enrollment | | -7.473 | 3.2822 | | -2.277 | | .024 | | -13.943 | | -1.003 | |  |
| Probability distribution: Normal Link function: Identity ^a^ | | | | | | | | | | | | |  |
| a. Target: Adherence | | | | | | | | | | | | |  |
| b. This coefficient is set to zero because it is redundant. | | | | | | | | | | | | |  |

| **Fixed Coefficients^a^** | | | | | | |
| --- | --- | --- | --- | --- | --- | --- |
| Model Term | Coefficient | Std. Error | t | Sig. | 95% Confidence Interval | |
|  |  |  |  |  | Lower | Upper |
| Intercept | 66.872 | 9.4122 | 7.105 | <.001 | 48.318 | 85.427 |
| burst=4 | -7.902 | 3.9521 | -1.999 | .047 | -15.693 | -.111 |
| burst=3 | -5.174 | 3.9531 | -1.309 | .192 | -12.966 | 2.619 |
| burst=2 | -12.355 | 3.1430 | -3.931 | <.001 | -18.551 | -6.159 |
| burst=1 | 0^b^ | . | . | . | . | . |
| Time between study start and participant enrollment | -6.797 | 3.0500 | -2.229 | .027 | -12.810 | -.785 |
| Reached study mid-point | 10.599 | 6.1501 | 1.723 | .086 | -1.525 | 22.723 |
| Did not reach study mid-point | 0^b^ | . | . | . | . | . |
| Duration of study participation | 19.226 | 9.0862 | 2.116 | .036 | 1.314 | 37.138 |
| Probability distribution: Normal  Link function: Identity^a^ | | | | | | |
| a. Target: Percentanswerspervisit_MoEv | | | | | | |
| b. This coefficient is set to zero because it is redundant. | | | | | | |
